# The Effect of Housing System and Gender on Relative Brain Weight, Body Temperature, Hematological Traits, and Bone Quality in Muscovy Ducks

**DOI:** 10.3390/ani12030370

**Published:** 2022-02-03

**Authors:** Ondřej Krunt, Adam Kraus, Lukáš Zita, Karolína Machová, Eva Chmelíková, Stanislav Petrásek, Petr Novák

**Affiliations:** 1 Department of Animal Science, Faculty of Agrobiology, Food and Natural Resources, Czech University of Life Sciences Prague, 165 00 Prague, Czech Republic; krunt@af.czu.cz (O.K.); krausa@af.czu.cz (A.K.); 2 Department of Genetics and Breeding, Faculty of Agrobiology, Food and Natural Resources, Czech University of Life Sciences Prague, Kamýcká 129, 165 00 Prague, Czech Republic; machovakarolina@af.czu.cz; 3 Department of Veterinary Sciences, Faculty of Agrobiology, Food and Natural Resources, Czech University of Life Sciences Prague, Kamýcká 129, 165 00 Prague, Czech Republic; chmelikova@af.czu.cz; 4 Department of Agricultural Machines, Faculty of Engineering, Czech University of Life Sciences Prague, Kamýcká 129, 165 00 Prague, Czech Republic; petrasek@tf.czu.cz (S.P.); novakpetr@tf.czu.cz (P.N.)

**Keywords:** alternative housing, body temperature, bone quality, relative brain weight

## Abstract

**Simple Summary:**

Free access to water with the possibility of swimming has the potential to be a good alternative to intensive housing of Muscovy ducks. The effect of this housing type was studied concerning hematological parameters, body temperature, relative brain weight, and bone quality. Birds with the possibility of swimming (S group) were compared to birds housed on deep litter with natural conditions (D group). Moreover, the effect of gender (G) was also studied. The housing of the birds had a significant effect on some hematological traits, body temperature, and relative brain weight. On the other hand, fracture toughness was not affected. Regarding the gender effect, it was found out that drakes had higher relative brain weight, lower body temperature, and higher fracture toughness of bones. These results help us understand the physiological and anatomical functioning of individual categories of animals monitored by us from a higher perspective with possible impacts on welfare and health.

**Abstract:**

The study was conducted during the summer season (June–August 2020). Two hundred sixty-four 5-week-old sexed Muscovy ducklings were randomly divided into four equal experimental groups by housing system and by gender. Each group had three replicates (22 birds/replicate) in a randomized design experiment. Regarding the hematological traits, the volume of leukocytes was higher in the D group (by 0.34 × 10^9^/L; *p* < 0.05) than in the S group. Furthermore, body temperature was found to be higher in ducks (by 0.84 °C; *p* < 0.05) and in the D group (by 0.5 °C; *p* < 0.05) in comparison with drakes and birds from the S group. Considering relative brain weight, drakes had higher values than ducks (by 0.56 g; *p* < 0.05), and birds from the S group also manifested higher values (by 0.78 g; *p* < 0.05). In terms of bone quality, there were no differences in studied parameters of tibia and femur bones regarding housing systems. The results provide valuable evidence of differences in the fattening of intensively bred Muscovy ducks within the housing system but also regarding gender.

## 1. Introduction

Pekin, Muscovy, and Mule ducks are commonly reared for meat production in Europe. There are large differences in housing systems, which are related to different behavior and levels of welfare [1]. The tendencies of improving a duck’s welfare are more and more common. Specifically, although outdoor runs were commonly used in the past [2], nowadays, different strategies of approving access to outdoor water for ducks are made to improve their health status or well-being [3]. Water provision in the form of swimming ponds allows ducks to manifest their species-specific behavior, such as dabbling, bathing, and swimming. Moreover, water effectively solves hygiene problems with dirty feathers and increases preening as the comfortable behavior of ducks [1]. Rearing ducks outdoor during the summer season can affect their body temperature, which can increase during hot days, or their blood profile [3]. The heat stress causes a reduction in feed intake and appetite and therefore compromises ducks’ welfare. These negative aspects can be eliminated by enriching the duck’s environment with access to water, which increases bird comfort [4]. The authors of [5] defined enrichment as an improvement in the biological functioning of captive animals resulting from modifications of their environment. Enrichment should be used for reducing negative emotional states such as fear, the stress associated with exposure to novel stimuli or boredom, and apathy from inappropriate housing. Moreover, environmental stimuli were found to increase the brain weight of rabbit males [6], probably due to stimulating neurogenesis in the hippocampus [7] or the higher energy requirements of animals [8]. Changes in brain size can also be supported by the expensive-tissue hypothesis, which predicts that the higher the brain size, the lower the size of another costly organ, such as the gut or others [9]. Additionally, the ability to learn tasks is a stimulus of increasing brain size [10]. Another factor that influences the well-being of animals, is bone quality, which can be affected by movement. In the study of [11], it was found that hens that were housed in floor systems with an increased possibility of movement had higher fracture toughness than hens in cages. In addition, the importance of the gender effect should not be overlooked. The differences between genders were described in intensively reared broiler chickens [12,13] due to hormonal differences [14]. Our overarching hypothesis is that free access to swimming ponds during the summer season improves Muscovy ducks’ welfare. Specifically, we hypothesize that outdoor runs with the possibility of swimming will increase the relative brain weight of birds. We also hypothesize the possibility of swimming will reduce body temperature, positively change blood profile, and improve the bone quality of birds. We further hypothesize that drakes will have higher relative brain weight, lower body temperature, changed blood profile, and higher fracture toughness of bones than ducks.

## 2. Materials and Methods

### 2.1. Animals and Husbandry

The present study was approved by the ethics committee of the Czech University of Life Sciences Prague (case number, 07/2020). The study was conducted during the summer season (June–August 2020). Two hundred and sixty-four 5-week-old sexed Muscovy ducklings were randomly divided into 4 equal experimental groups by housing system and by gender (female/deep litter, male/deep litter, female/swimming pond, male/swimming pond). Each group (66 birds per group and gender) had three replicates (22 birds/replicate). Birds were housed in a close-sided house on deep litter (D) with regard to gender and in an open-sided house with free access to a swimming pond (S) with regard to gender under natural conditions. On average, the length of the day was 16 h and that of the night 8 h. Moreover, the average temperatures were: 17.9 °C (June), 18.9 °C (July), and 20.3 °C (August). Housing systems for S groups included trees near the swimming ponds, which provided shadow during hot summer days. All animals were reared under the same conditions. Wheat straw was used as deep litter in every housing system. Each group was kept at density of 4 ducks per m^2^. Moreover, group S had a swimming pond (10 m length × 6 m width × 3 m depth with concrete floor) at its disposal. Fresh water was provided into the pond from supply channels. All birds were fed ad libitum with a pelleted diet (20% CP and 11.2 MJ/kg ME) and had water fully available in their housing system. At the end of the experiment (14 weeks of age), all birds were slaughtered by jugular venesection after 12 h fasting.

### 2.2. Measurements of Hematological Parameters

Blood samples from 9 animals (14 weeks of age) from each group and replicates were taken during slaughtering in sterile syringes from the jugular vein. Samples were centrifuged at 2.328× *g* for 15 min at 4 °C to collect serum. After obtaining whole blood samples, blood films were made using the slide method of [15]. Blood films were stained using Pappenheim May–Grunwald Giemsa stain. A differential number of leukocytes was made of three horizontal edge fields followed by two fields towards the center. They were followed by two fields in a horizontal direction and after that by two fields in a vertical direction to obtain the edge again. The field takes a crisscross shape with right angles. Two hundred cells, with 100 cells on each edge of the film, were differentiated, and the percentages of heterophils and lymphocytes were calculated. Erythrocytes were determined manually by hemocytometer. Blood hemoglobin (Hb) was determined according to [16]. Mean corpuscular volume (MCV) and mean corpuscular hemoglobin concentration (MCHC) were calculated according to [17]. The H/L ratios were determined according to the formula:

H/L = heterophiles/lymphocytes.

### 2.3. Body Temperature

The body temperature from 9 animals from each group and replicate was recorded from rectum by thermometer (TH—802, OEM brands, CE ISO FDA, Guangdong, China) once a week on Wednesday from 12:00 until 12:30. All birds were gently treated, and the thermometer was tenderly inserted into the rectum to 2 cm depth. The temperature was recorded after the alarm signal (usually after 45 s). Each animal was processed for less than 1.5 min.

### 2.4. Relative Brain Weight

At 14 weeks of age, all birds (66 birds/group/gender) were slaughtered by cutting the jugular vein. All brains were removed according to the methods of Bozicovich et al. [6] by cutting the frontal bone with stainless steel scissors, and they were weighed on an analytical scale Ohaus (Model: Traveler TA502, Parsippany, NJ 07054) with 0.01 g precision. Housing system and gender averages were used in the analyses.

### 2.5. Bone Quality Characteristics, Sampling, and Analyses

The raw bones from 9 animals from each group and replicate were examined for weight, length, width and fracture toughness. Bones were weighed on an analytical scale Ohaus (Model: Traveler TA502, Parsippany, NJ, USA, 07054), and diameter was measured by dial caliper (±0.02 mm) at the mid-diaphysis, where the breaking point was. Femur and tibia bones of the left hind leg were taken at slaughter and individually packed in polyethylene bags and stored at −20 °C until the analysis, when they were thawed overnight. When fully defrosted, soft tissue was removed from the tibia and femur. The length of the tibia/femur was measured as the distance from tibia/femur spine to inferior articular surface by dial caliper (±0.02 mm). The tibia and femur were subsequently boiled for 15 min in 95 °C water, de-fleshed and de-fatted, and dried at 25 °C for 24 h. The breaking strength was determined with a three-point flexure test using a Instron^®^ Model 3342 (Instron, Norwood, MA, USA), and the load rate was 30 mm/min. The space between the two fulcra points supporting the bones was 45 and 38 mm. The bones were continually oriented for examination with their natural convex shape downwards.

#### Statistical Analyses

The effect of gender and housing system on each hematological trait, body temperature, relative brain weight, and bone quality parameters was assessed by the mixed model using the MIXED procedure of SAS (SAS Institute Inc., Cary, NC, USA, 2011):

yijk = µ + HSi + Gj + (HS×G) ij + eijk,

where yijk is the value of trait, µ is the overall mean, HSj is the effect of the housing system, Gi is the effect of gender (HS × G) ij is the effect of the interaction between housing system and gender, and eijk is the random residual error. The significance of the differences among groups was tested by Duncan’s multiple range test. The value of *p* ≤ 0.05 was considered as significant for all measurements.

## 3. Results

### 3.1. Relative Brain Weight

**Figure 1:**
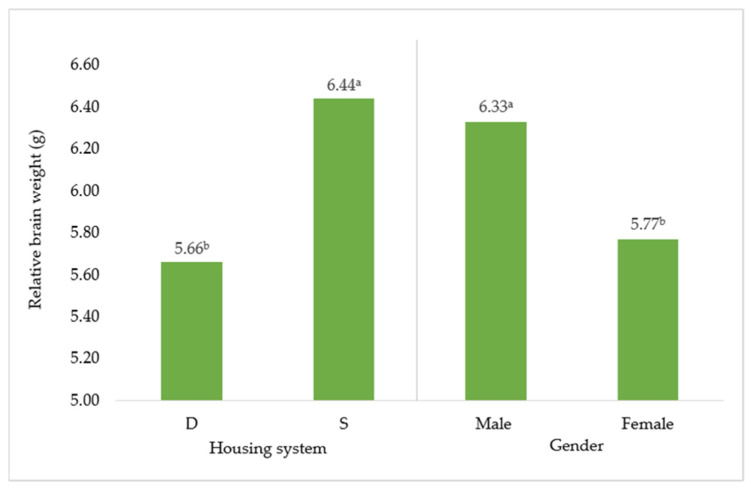
Effect of housing system and gender on relative brain weight (g). D = deep litter; S = swimming ponds. ^a,b^ Different superscripts indicate significant differences between means (*p* ≤ 0.05).

The effect of housing system and gender of Muscovy ducks on relative brain weight is displayed in Figure 1. Considering relative brain weight, differences were between gender (by 0.56 g; *p* < 0.05) and housing system (by 0.78 g; *p* < 0.05).

### 3.2. Hematological Parameters and Body Temperature

**Figure 2:**
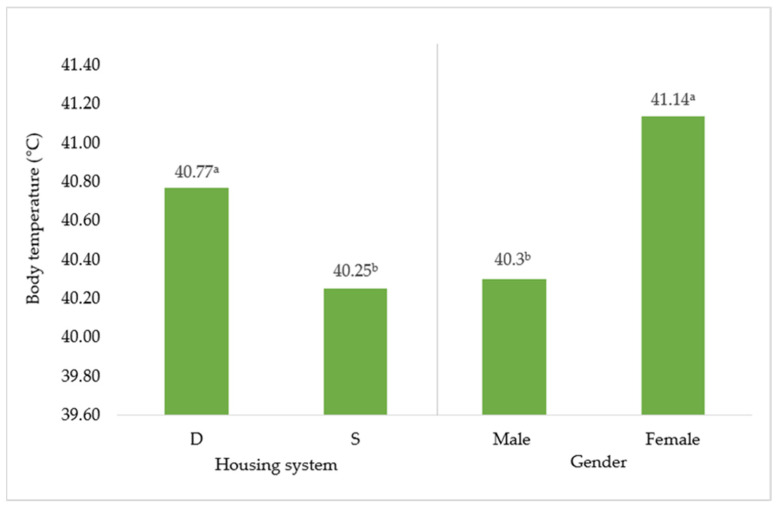
Effect of housing system and gender on body temperature (°C). D = deep litter; S = swimming ponds. ^a,b^ Different superscripts indicate significant differences between means (*p* ≤ 0.05).

The results concerning the hematological traits of birds are presented in Table 1, and body temperature values are displayed in Figure 2. Statistically significant interactions are discussed in detail in the text but not described in tables. Regarding the hematological traits, volume of leukocytes was higher in the D group (by 0.34 × 10^9^/L; *p* < 0.05). Moreover, significant interaction between HS and G was found for leukocytes, where the highest values had drakes from the D group (25.07 × 10^9^/L; *p* < 0.05) and the lowest values had ducks from the S group, the D group, and drakes from the S group (24.58, 24.42, and 24.21 × 10^9^/L; *p* < 0.05, respectively). Values of lymphocytes tended to be higher also in the D group in comparison with the S group). Furthermore, body temperature was found to be higher in ducks (by 0.84 °C; *p* < 0.05) and in the D group (by 0.5 °C; *p* < 0.05).

### 3.3. Bone Quality

In terms of bone quality (Table 2), there were no differences in studied parameters of tibia and femur bones regarding to housing systems. There was found just a tendency of higher weight in femur bones in favor of the D group (by 0.32 g; *p* < 0.05). On the contrary, a significant effect of gender was found in every evaluated parameter of both bones due to sexual dimorphism between the gender in favor of drakes.

## 4. Discussion

### 4.1. Relative Brain Weight

Considering relative brain weight, differences between gender could seem expected due to sexual dimorphism between ducks and drakes, which are substantially larger than ducks [1]. However, the results may have a different reason. In humans, there was confirmation of the sex difference in adult brain size [18] or in 18-year-old students’ brains [19]. Pakkenberg and Gundersen [20] explained the differences using a higher number (4 billion more) of cortical neurons in men. More exceptional are the differences in groups, which were housed differently. In total, birds housed in a system with a swimming pond had heavier brains than birds from the deep litter. According to the scientific literature, there are several studies that have found differences in relative brain weight among the birds. For example, parrots have larger brains as a response to higher seasonality and precipitation [21]. Passerine birds have larger brains when they experience higher environmental variation by migrating [22]. Unfortunately, there are no studies of intensively housed ducks or geese that considered relative brain weight as a possible aspect of physiological or mental state. The reason could be linked with higher interaction with the environment and connected exploratory behavior, since providing environmental stimuli influences brain weight in mice [10] and in male rabbits [6]. The authors of the latter study postulate that environmental enrichment promotes the development of specific regions in rabbits’ brains. They base their claims on the conclusions of the study of [23], which found increased cortical thickness and enhanced dendritic ramification in the brains of rodents, which were exposed to the presence of environmental enrichment. In addition, our hypothesis could be supported by the findings of [24], which reported significant decreases in absolute and relative brain volume in captive-bred waterfowl compared to their wild counterparts. Additionally, the decrease in brain size in domesticated waterfowls in comparison with wild animals is generally attributed to a decrease in functional requirements resulting from the unnatural environment. Our results could indicate that the environment had a strong influence on brain development in Muscovy ducks.

### 4.2. Hematological Parameters and Body Temperature

The current study results show a decrease in leukocytes and lymphocytes in the S group compared to the D group, which could be attributed to potential non-specific immune response induced by heat [25], which probably acted more in deep litter housing. On the contrary, in the study of [3], it was reported that the highest volume of lymphocytes was in birds with the longest time with access to water. The body temperature of birds is also related to the time spent on the water. Animals from the S group had lower body temperature than those from the D group. This trend was also confirmed by [26] and by [3] with the explanation of cooling the body due to better evaporation. More interesting are the differences between ducks and drakes, which had lower body temperatures than ducks. Pis [27] mentioned the link between metabolic rate and body temperature in galliform birds, that body temperature is associated with the large variability because of gender, season, and measurement conditions and therefore resulted in “unpopularity”. The effect of gender on body temperature was previously studied in mice. The study of [28] reported the importance of body temperature measurements at the same time of the day when it is performed. This is consistent with our methodology of measuring body temperature at the same time of the day. Findings of this study reported that female mice had higher body temperature than males, with a possible effect on lifespan. The activity of hormones is suggested as one possible explanation for the higher body temperature in females. In general, progesterone promotes less vasodilatation, heat conservation, and higher values of body temperature in women [29]. In poultry, the effect of gender on body temperature was also confirmed in Japanese quails. Female quails had higher body temperature than male quails [30]. It is very difficult to explain the differences between genders, but another possible connection to consider could be the basal metabolic rate, which was linked with increased capacity of heat production [31].

### 4.3. Bone Quality

In general, bone fracture toughness can reflect the welfare levels of animals in their housing system. Fractures of keel bones are a real problem in rearing systems of intensive laying hens [32]. There were also found to be differences in fracture toughness due to higher movement in tibia bones of hens that were housed in flat floors (these hens had bones more resistant to fracture) than of hens in cages [11]. Results of these studies should mean that ducks that will have a greater ability to move will also have bones that are more resistant to fracture. On the other hand, our results show that birds from both systems did not differ in these terms. In the end, this is good information, because we can summarize that no-swimming housing conditions did not decrease bone strength or vice versa. Considering the gender effect, which was significant in all parameters of tibia and femur bones, the length, width, and weight of these bones were expectably higher in drakes due to their weight dimorphism in general. The fracture toughness was probably also affected by the same factor, which was mentioned in the previous statement. It was higher in drakes due to greater width or weight, which should indicate the higher content of elements that affected bone strength [33].

## 5. Conclusions

In conclusion, free access to water with the possibility of swimming had an effect on leukocytes and a positive effect on body temperature. Moreover, relative brain weight was strongly influenced by housing, whereas bone quality did not differ. With regard to gender, no effect on hematological traits was found, whereas body temperature was significantly higher in ducks when compared to drakes. Additionally, ducks had a lower relative brain weight than drakes. Nevertheless, according to the bone quality analyses, drakes had higher values of every single parameter than ducks. Our results provide valuable evidence of differences in the fattening of intensively bred Muscovy ducks within the housing system, but also regarding gender. These results reveal the physiological and anatomical functioning of individual categories of animals monitored by us from a higher perspective with possible impacts on welfare and health.

## Figures and Tables

**Table 1:** The effect of housing system and gender on hematological traits.

Traits	Housing System (HS)		Gender (G)		SEM	*p*-Value		
	D	S	Male	Female		HS	G	HS × G
Hematocrit (%)	41.80	42.11	41.12	42.81	0.009	0.8620	0.3514	0.1102
Hemoglobin (g/L)	133.06	134.61	131.34	136.3	2.886	0.7896	0.3905	0.1135
Erythrocytes (10^12^/L)	2.90	2.93	2.86	2.97	0.063	0.7813	0.3965	0.1160
Leukocytes (10^9^/L)	24.74 ^a^	24.40 ^b^	24.64	24.51	0.096	0.0445	0.4113	0.0040
Heterophiles (10^9^/L)	10.82	11.10	11.06	10.87	0.109	0.1897	0.3628	0.0888
Lymphocytes (10^9^/L)	15.67	15.30	15.46	15.12	0.010	0.0512	0.7607	0.4484
H/L ratio	0.69	0.72	0.71	0.72	0.018	0.6541	0.4589	0.3461
MCV (fL)	144.21	143.65	143.67	144.18	0.213	0.1986	0.2440	0.8395
MCHC (g/L)	318.34	319.42	319.37	318.40	0.477	0.2709	0.3233	0.9631

D = deep litter; S = swimming ponds; MCV = mean corpuscular volume; MCHC = mean corpuscular hemoglobin concentration. ^a,b^ Different superscripts within a row indicate significant differences between means (*p* ≤ 0.05).

**Table 2:** The effect of housing system and gender on some functional parameters of tibia and femur bones.

Traits	Housing System (HS)		Gender (G)		SEM	*p*-Value		
	D	S	Male	Female		HS	G	HS × G
*tibia*								
Length (mm)	112.32	114,09	124.99 ^a^	102.21 ^b^	2.125	0.2826	0.0001	0.2604
Width (mm)	8.03	8.05	9.20 ^a^	6.95 ^b^	0.220	0.7561	0.0001	0.5233
Weight (g)	10.64	10.63	14.65 ^a^	6.88 ^b^	0.721	0.3454	0.0001	0.5233
Fracture toughness (N/cm^2^)	386.29	379.34	488.04 ^a^	283.95 ^b^	19.925	0.3564	0.0001	0.9206
*femur*								
Length (mm)	69.26	69.78	75.00 ^a^	62.04 ^b^	1.380	0.4303	0.0001	0.8159
Width (mm)	9.34	9.40	10.62 ^a^	8.13 ^b^	0.247	0.7647	0.0001	0.5152
Weight (g)	7.90	7.58	10.44 ^a^	5.04 ^b^	0.496	0.1008	0.0001	0.5542
Fracture toughness (N/cm^2^)	372.22	361.63	450.17 ^a^	283.68 ^b^	17.716	0.5925	0.0001	0.3345

D = deep litter; S = swimming ponds. ^a,b^ Different superscripts within a row indicate significant differences between means (*p* ≤ 0.05).

## Data Availability

The data presented in this study are available by reasonable request from the corresponding author.
